# DCSE:Double-Channel-Siamese-Ensemble model for protein protein interaction prediction

**DOI:** 10.1186/s12864-022-08772-6

**Published:** 2022-08-04

**Authors:** Wenqi Chen, Shuang Wang, Tao Song, Xue Li, Peifu Han, Changnan Gao

**Affiliations:** 1 College of Computer Science and Technology, China University of Petroleum (East China), Qingdao, China; 2 Department of Artificial Intelligence, Polytechnical University of Madrid, Madrid, Spain

**Keywords:** Protein-protein interaction, Ensemble based network, Siamese based network

## Abstract

**Background:**

Protein-protein interaction (PPI) is very important for many biochemical processes. Therefore, accurate prediction of PPI can help us better understand the role of proteins in biochemical processes. Although there are many methods to predict PPI in biology, they are time-consuming and lack accuracy, so it is necessary to build an efficiently and accurately computational model in the field of PPI prediction.

**Results:**

We present a novel sequence-based computational approach called DCSE (Double-Channel-Siamese-Ensemble) to predict potential PPI. In the encoding layer, we treat each amino acid as a word, and map it into an N-dimensional vector. In the feature extraction layer, we extract features from local and global perspectives by Multilayer Convolutional Neural Network (MCN) and Multilayer Bidirectional Gated Recurrent Unit with Convolutional Neural Networks (MBC). Finally, the output of the feature extraction layer is then fed into the prediction layer to output whether the input protein pair will interact each other. The MCN and MBC are siamese and ensemble based network, which can effectively improve the performance of the model. In order to demonstrate our model’s performance, we compare it with four machine learning based and three deep learning based models. The results show that our method outperforms other models in all evaluation criteria. The Accuracy, Precision, $F_{1}$, Recall and MCC of our model are 0.9303, 0.9091, 0.9268, 0.9452, 0.8609. For the other seven models, the highest Accuracy, Precision, $F_{1}$, Recall and MCC are 0.9288, 0.9243, 0.9246, 0.9250, 0.8572. We also test our model in the imbalanced dataset and transfer our model to another species. The results show our model is excellent.

**Conclusion:**

Our model achieves the best performance by comparing it with seven other models. NLP-based coding method has a good effect on PPI prediction task. MCN and MBC extract protein sequence features from local and global perspectives and these two feature extraction layers are based on siamese and ensemble network structures. Siamese-based network structure can keep the features consistent and ensemble based network structure can effectively improve the accuracy of the model.

**Supplementary Information:**

The online version contains supplementary material available at 10.1186/s12864-022-08772-6.

## Background

Protein-protein interaction (PPI) has always occupied an important position in the field of proteomics science because it plays a key role in the foundation of biological processes. The traditional research of PPI was mainly carried out by laboratory-based experimental techniques, such as yeast two-hybrid screening [1], X-ray crystallography, protein chips, and affinity purification [2] [3]. These methods have been used to study PPI at the molecular level and have generated a substantial amount of data about potentially interacting protein pairs, but these biologically based methods are time-consuming and expensive [3]. To solve these disadvantages, computational and deep learning based methods have gradually begun to be applied to the field of predicting PPI.

In recent years, many new methods have been proposed to make breakthroughs in the field of PPI. These deep learning based studies can be roughly divided into three categories [4] according to the representation of proteins: protein structure-based, sequence-based and both of all.

Previous studies have shown that only using protein sequence information can predict PPI tasks well [4–6]. In these studies, the encoding process of the protein sequence mainly relies on the position-specific scoring matrix (PSSM), Conjoint Triads (CT), Auto-Covariance (AC), and Local Descriptor (LD). Although these four encoding methods have shown ability in the PPI prediction task, there are still some disadvantages. Previous studies [7] have pointed out that getting PSSM matrix is time-consuming, so it is almost impossible to perform PSSM encoding on large-scale datasets. CT, AC, and LD use 343, 210 and 630 dimensional vectors to represent the feature of a protein separately. However, these features are all about the physicochemical features of amino acids. In some other works [8, 9], one-hot vectors are used to encode different amino acids, but the shortcoming is that the sparse feature matrix of one-hot encoding consumes memory.

Recently, natural language processing technology(NLP) has developed rapidly. Considering that the amino acid sequence is similar with language sentences, some researchers [10–15] have performed NLP technology on PPI tasks and obtained good results. Inspired by Nikhil et al’s work [12], we treat each amino acid as a word and map it into an N-dimensional vector, so the amino acid with length L can be encoded into a matrix with the size of L x N, where L is the length of protein sentence, and N is the encoding vector dimension of each amino acid.

In recent years, the siamese network and ensemble network performed well in the field of deep learning. Yiwei Li’s work [4] utilizes CNN and RNN together to extract protein sequence information from two perspectives to compose an ensemble network. Xiaodi Yang’s work [16] employs a siamese CNN architecture with two identical CNN sub-networks that share the same parameters to capture complex relationship between two proteins.

In this paper, we present a novel sequence-based computational approach called DCSE (Double-Channel-Siamese-Ensemble) to predict potential PPI. Specifically, we treat the amino acid as a word and map the word into an N-dimension vector. Then we employ Multilayer Convolutional Neural Network (MCN) and Multilayer Bidirectional Gated Recurrent Unit with Convolutional Neural Networks (MBC) to extract the protein feature from two perspectives. MCN can further extract local features of protein sequences by setting small kernel sizes. MBC aims to extract the protein sequence information from the global perspective by extracting the protein sequence features from left-to-right and right-to-left simultaneously. The MCN and MBC are siamese and ensemble-based networks. We show the superiority of our model performance by comparing it with four machine learning based methods and three deep learning based methods, and conduct ablation experiments to analyze the importance of different components in DCSE model.

## Result

### Dataset

The original data comes from String v11 [17] dataset. There are 8397 protein sequences hence it can compose $8397^2$ protein pairs. All the sequences are homo sapiens proteins. However, there are only 117,953 protein pairs that are verified protein protein interaction pairs. How select the negative samples is always important in PPI prediction field because there are some pairs that indeed interact with each other but haven’t been verified by the biological experiments. To guarantee the credibility of the negative samples, we use the Dissimilarity Threshold [18] method to select the negative samples. The core idea of the Dissimilarity Threshold is that if sequence A and sequence B share more than 40% similarity, and sequence A and sequence C are verified as interaction pairs, then sequence B and sequence C is not considered the negative sample. The idea behind this method is that two proteins have higher similarities, they are more likely to be similar in their 3D structure. Note that PPI occurs in the 3D structure, so two proteins that are similar in the 3D structure tend to interact with the same protein. So we first remove the sequence that shares similarities of more than 40% by CD-HIT [19] to guarantee the sequences are not homologous. After this step, there are only 7390 protein sequences remaining and there are only 65,232 verified interaction protein pairs. After removing the homologous sequences, we use random sampling to get 65,232 negative samples. So our dataset has 7390 proteins, 65,232 positive samples and 65,232 negative samples. We follow the 7:3:1 division ratio to divide all the dataset into training set, validation set, and testing set. In addition, previous work [20] pointed out that the PPI prediction is an inherently imbalanced problem, so we make up another three 1:3, 1:5, and 1:10 imbalanced datasets to further explore the performance of our method in the imbalanced situation.

### Evaluation Metrics

The prediction of PPI is regarded as a binary classification task. In order to evaluate the model comprehensively, we adopt five evaluation metrics, including Accuracy (Acc), Precision (Pre), Recall, $F_{1}$ score and MCC. Accuracy is the percentage of correct predictions. Precision reflects the quality of a positive prediction. Recall is the measure of our model correctly identifying true positives. $F_{1}$ score and MCC consider the performance on the positive samples and the negative samples together. Their calculation formula is as follows.

1 $$\begin{aligned} \mathrm {Accuracy}=\frac{T P+T N}{T P+T N+F P+F N} \end{aligned}$$

2 $$\begin{aligned} \text{ Precision } =\frac{T P}{T P+F P} \end{aligned}$$

3 $$\begin{aligned} \text{ Recall } =\frac{T P}{T P+F N} \end{aligned}$$

4 $$\begin{aligned} F_{1}=\frac{2 \times \text{ Precision } \times \text{ Recall } }{ \text{ Precision } + \text{ Recall } } \end{aligned}$$

5 $$\begin{aligned} \text{ M } C C=\frac{T P \times T N-F N \times F P}{\sqrt{(T P+F P) \times (T P+F N) \times (T N+F P) \times (T N+F N)}} \end{aligned}$$

Where true positives (TP) denote the number of interacting PPI identified correctly. True negatives (TN) denote the non-PPI identified correctly. False positives (FP) denote the number of incorrectly predicted PPI. False negatives (FN) denote the number of incorrectly predicted non-PPI.

### Implementation

Our model is implemented with Pytorch-1.9.0 and trained with GeForce RTX 3090 GPU support. We perform 5-fold cross-validation and record the average of the 5-fold cross-validation and the standard deviations. The main hyperparameters are explored with grid search, which is used to get the optimal hyperparameters. The hyperparameters and the optimal values are shown in Table 1.

**Table 1:** The optimal value of hyperparameters

Learning rate	0.01
Batch size	64
Activation function	LeakReLu(0.3)
Dropout rate	0.2
Weight initialization	uniform
Weight regularization	L2
Optimizer	Adam
kernel size	3
$\beta$	0.5

### Comparison of the model performance

**Fig. 1:**
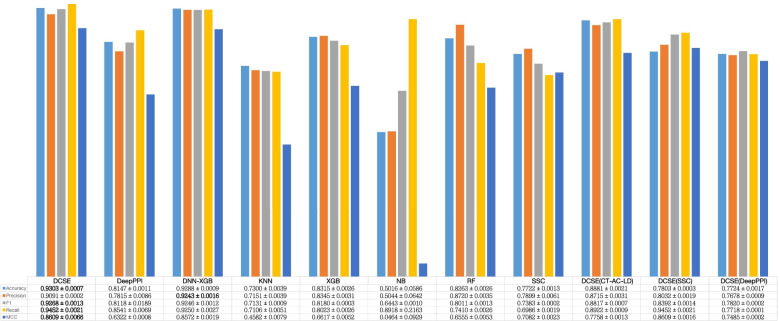
The comparison results of the eight models

To further verify the performance of our model, we compared it with other current deep learning methods including SSC [21], DNN-XGB [22], DeepPPI [23], and traditional machine learning methods including Naive Bayes (NB) [24], K-nearest neighbors with K=5 (KNN) [25], Random Forest (RF) [26], XGBoost [27]. The selection criteria for these deep learning based models are that they are all sequence-based, and the source code can be obtained. The innovation of SSC is that it proposes a new encoding method, and finally turns a pair of protein sequences into a $(1800 + 1800) \times 3$ matrix with 2D CNN to further extract the feature. DNN-XGB uses the CT-AC-LD encoding method to extract features through DNN (Deep-neural-network) and finally passes through the XGB classifier. When we retrained the DNN-XGB model, the phenomenon of under-fitting occurs, that is because there are too many drop out layer, so we cancel the dropout layer in DNN used in the original code, and only keep the dropout layer in the prediction layer. DeepPPI employs deep neural networks to effectively learn the representations of proteins from common protein descriptors such as Amino Acid Composition (AAC), DipeptideComposition (DC), Composition, Transition, Distribution, Quasi-sequence-order (QSOD). The comparative results are shown in Fig. 1.

According to Fig. 1, it is clear that the deep learning based methods are much better than the machine learning based methods. The Accuracy of machine learning based methods is from 0.5016 to 0.8315, while the Accuracy of deep learning based methods is from 0.7722 to 0.9303. In terms of deep learning methods, DCSE (Our) obtains the best performance among all the evaluation indicators. In addition, we also use the same protein features used in DNN-XGB called DCSE (CT-AC-LD), and the performance of DCSE (CT-AC-LD) is also shown in Fig. 1, from which we can see the performance of DCSE (CT-AC-LD) is obviously not as good as DCSE because all evaluation indicators of DCSE (CT-AC-LD) have declined. We also use SSC encoding method and the result is also shown in Fig. 1. The result shows that DCSE (SSC) is also not good as DCSE. Because the SSC encoding method only considers the statistical information of the amino acid such as the frequency of each amino acid, which is hard to fully reflect the information of the protein sequence. In addition, we also change our feature extraction layer to the one used in the DeepPPI and the result is shown in Fig. 1 called DCSE (DeepPPI). The result shows that our methods outperform the DCSE (DeepPPI). The reason is that DeepPPI ignores the global information of protein sequence, but the MBC layer is employed to extract the global information in our model. In addition, we record the confidence interval for all the methods, and conduct hypothetical test between DCSE and DNN-XGB, which can be seen in supplementary material.

**Fig. 2:**
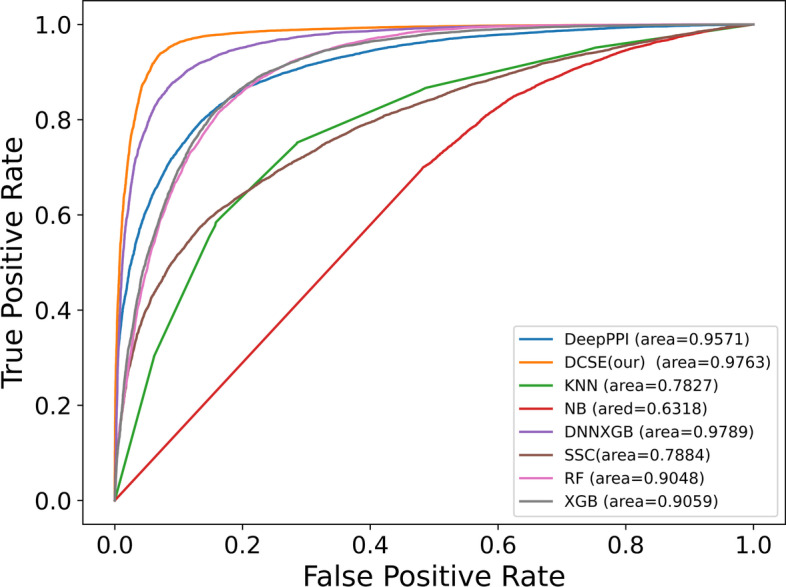
The AUC-ROC curves of different methods

**Fig. 3:**
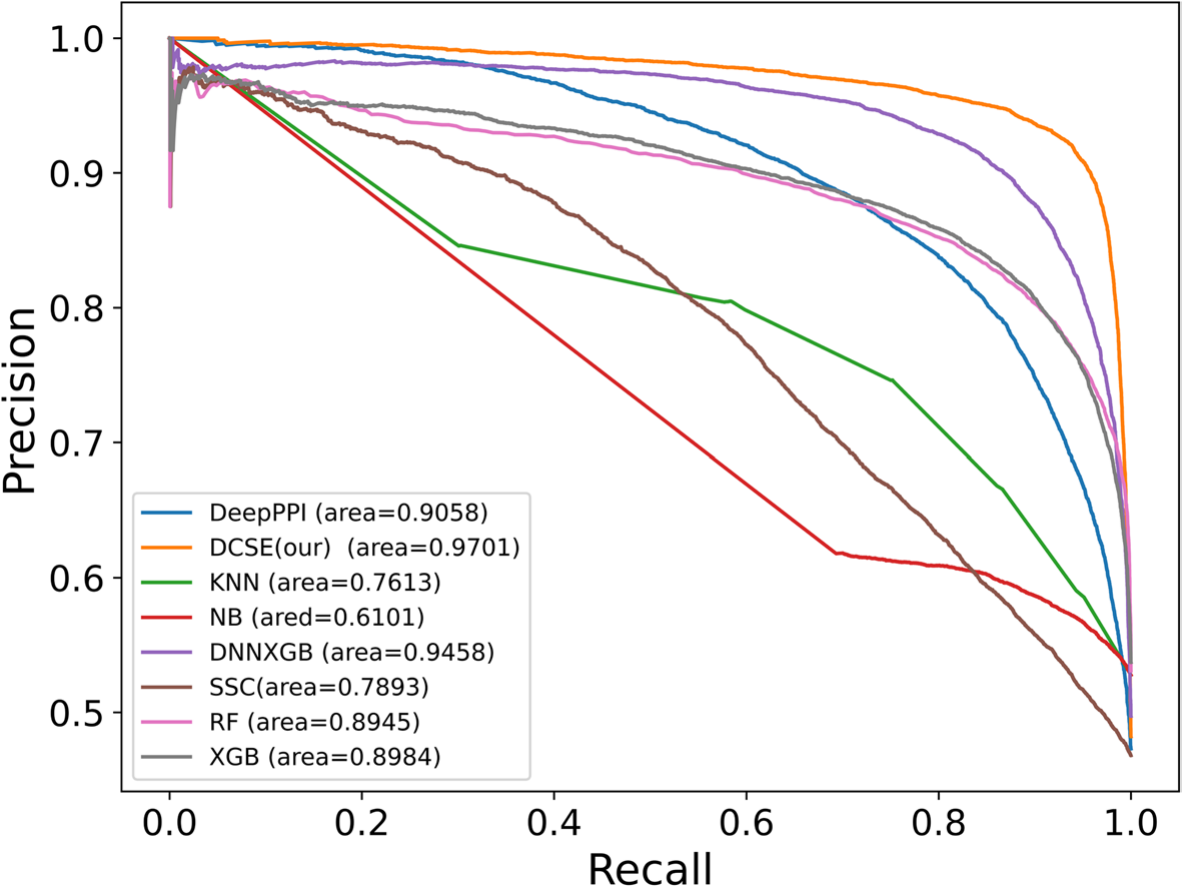
The Precision-Recall curves of different methods

We also plot the AUC-ROC curve and Precision-Recall curve as shown in Figs. 2 and 3. The area of AUC-ROC curve and Precision-Recall curve is 0.9763 and 0.9701, which is the best among all the methods.

### Comparison of the combination of different channels

**Fig. 4:**
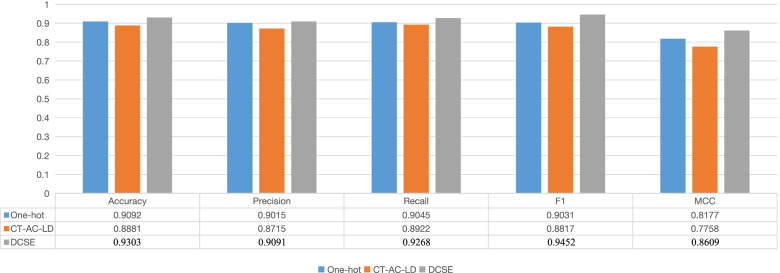
The impact of different channel combinations on PPI task

In order to get the complicated information of protein sequence, we design two channels to extract the local feature and global feature respectively. To explore the influence of each channel on PPI, we made a comparison for the combination of different channels. We build two reference models with single channel separately. The architecture except the channel setting of these two models is the same with our proposed model. The results are shown in Fig. 4, from which we can see that the performance of the combination of both two is better than any single one of them.

### Comparison of the combination of different $\beta$

**Fig. 5:**
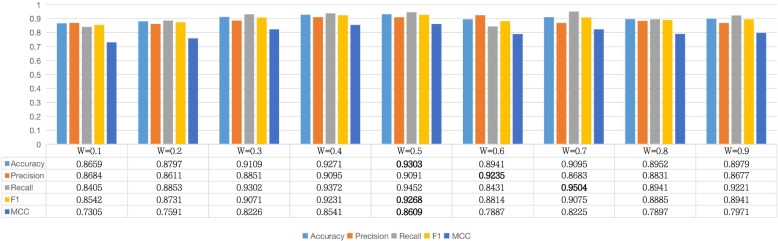
The effect of different $\beta$ values on the model performance

We have shown that using two channels can achieve better results, but what is the most suitable ratio of two channels to achieve the best performance is still need to explore. $\beta$ is the hyperparameter to balance the contribution of two channels. We designed a series of comparative experiments to explore the best value of the hyperparameter. We take it from 0.1 to 0. 9. The result of our experiment is shown in Fig. 5. Our results show that the metrics of Accuracy, $F_{1}$ and MCC all achieve the best value when $\beta$ is set to 0.5, which means that the two channels have the same contribution on the PPI task.

### Comparison of different value of K and N

K determines how many contiguous amino acids as the smallest unit, and N determines how many dimensions this smallest unit is mapped to. More details can be found in section 4.1. We test K in [1,2,3,4] and N in [20,25,30]. Note that when the K exceeds 5, there are too many different amino acid combinations($22^5$), which will greatly increase the training time and memory consumption. Then we get 3*4 different hyperparameter combinations. This experiment is performed under the $\beta$=0.5 and the result of our experiment is shown in Table 2. The model achieves the best result when N is 25 and K is 1 because Accuracy, $F_{1}$score, Recall, MCC are all highest among all the different K and N.

**Table 2:** The effect of different the K and N

	ACC	Pre	$F_{1}$	Recall	MCC
N=20&K=1	0.8905±0.0009	0.8491±0.0007	0.8883±0.0011	0.9312±0.0013	0.7846±0.0021
N=20&K=2	0.8884±0.0007	**0.9201±0.0019**	0.8747±0.0005	0.8337±0.0017	0.7777±0.0017
N=20&K=3	0.8758±0.0010	0.8739±0.0014	0.8659±0.0007	0.8581±0.0013	0.7504±0.0032
N=20&K=4	0.8751±0.0008	0.8735±0.0009	0.8651±0.0010	0.8569±0.0009	0.7491±0.0048
N=25&K=1	**0.9303±0.0007**	0.9091±0.0002	**0.9268±0.0013**	**0.9452±0.0021**	**0.8609±0.0066**
N=25&K=2	0.9172±0.0011	0.8958±0.0005	0.9131±0.0018	0.9311±0.0015	0.8347±0.0027
N=25&K=3	0.9021±0.0014	0.8645±0.0007	0.8994±0.0010	0.9372±0.0021	0.8068±0.0009
N=25&K=4	0.9137±0.0003	0.8912±0.0010	0.9095±0.0009	0.9287±0.0020	0.8277±0.0018
N=30&K=1	0.9101±0.0032	0.8617±0.0009	0.9091±0.0021	0.9619±0.0019	0.8255±0.0039
N=30&K=2	0.9071±0.0007	0.8902±0.0021	0.9018±0.0019	0.9137±0.0024	0.8138±0.0015
N=30&K=3	0.8981±0.0005	0.8667±0.0007	0.8945±0.0008	0.9241±0.0019	0.7978±0.0014
N=30&K=4	0.9031±0.0029	0.8767±0.0018	0.8989±0.0021	0.9223±0.0009	0.8069±0.0020

### Comparison of different encoding method

**Fig. 6:**
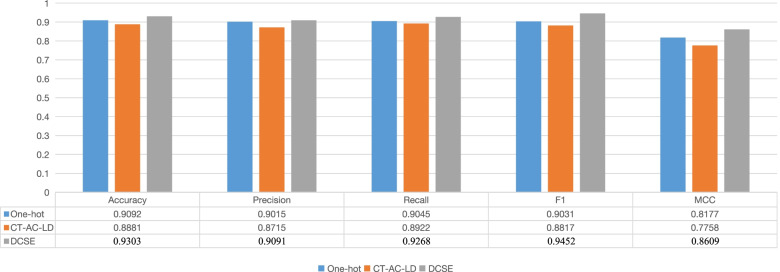
The effect of different encoding methods on the results

To verify whether our encoding method is effective, we choose the currently widely used one-hot and CT-AC-LD encoding to verify that our encoding method is effective. Specifically, the input of one-hot vector encoding is a 1000*22 matrix. For CT-AC-LD, the combined input dimension is 1183(343+210+630), we use 0 as padding, change the input dimension to 1200, and then resize it to 40*30 as the input of the model. The result of our experiment is shown in Fig. 6, from which we can see our encoding method achieves the best performance among the three encoding methods.

### Performance on the imbalanced data

In order to simulate the real situation as much as possible, we constructed three imbalanced datasets with positive and negative sample ratios of 1:3, 1:5, and 1:10, respectively. The experimental results are shown in Tables 3, 4, and 5. As the ratio of negative to positive samples increases, the accuracy of almost all models increases, but this is meaningless because the accuracy cannot accurately reflect the performance of the model on an imbalanced dataset. We focus more on $F_{1}$ score and MCC. Both of them comprehensively consider the prediction effect of the model on positive samples and negative samples. The final result proves that our model is less affected by imbalanced data, The $F_{1}$ score on the three imbalanced datasets is 0.9132, 0.9088, 0.9033, and the MCC is 0.8844, 0.8904, 0.8935. However, $F_{1}$ socre and MCC of all other models decreased with increasing negative samples. Taking DNN-XGB as an example, the values of $F_{1}$ score on three imbalanced datasets are 0.8994, 0.8703, 0.7994, and the values of MCC on three imbalanced datasets are 0.8672, 0.8467, 0.7865.

**Table 3:** The performance on the 1:3 imbalanced dataset

	ACC	Pre	$F_{1}$	Recall	MCC
DCSE	**0.9565±0.0072**	0.9012±0.0123	**0.9132±0.0021**	**0.9256±0.0241**	**0.8844±0.0131**
DeepPPI	0.8696±0.0057	0.7293±0.0184	0.7456±0.0122	0.7639±0.0312	0.6243± 0.0408
DNN-XGB	0.9507± 0.0019	**0.9194±0.0033**	0.8994±0.0123	0.8803±0.0055	0.8672±0.0052
KNN	0.7825±0.0026	0.5815±0.0071	0.5185±0.0032	0.4678±0.0047	0.3838± 0.0064
XGB	0.8813±0.0016	0.8253± 0.0009	0.7379±0.0031	0.6672±0.0071	0.6686±0.0049
NB	0.3387±0.1767	0.2804±0.0602	0.4267±0.0503	0.8928± 0.2143	0.0444±0.0888
RF	0.8690±0.0014	0.9148± 0.0019	0.6675±0.0122	0.5255 ± 0.0053	0.6286± 0.0045
SSC	0.8234±0.0017	0.6419±0.0021	0.6363±0.0019	0.6440± 0.0117	0.5242± 0.0043

**Table 4:** The performance on the 1:5 imbalanced dataset

	ACC	Pre	$F_{1}$	Recall	MCC
DCSE	**0.9693±0.0013**	0.9015±0.0003	** 0.9088±0.0002**	**0.9163±0.0035**	**0.8904±0.0022**
DeepPPI	0.9052±0.0046	0.7300±0.0281	0.7087±0.0094	0.6901±0.0022	0.6532±0.0124
DNN-XGB	0.9584±0.0003	**0.9079±0.0006**	0.8703±0.0014	0.8358±0.00425	0.8467± 0.0014
KNN	0.8334±0.0006	0.5017±0.0027	0.4043±0.0132	0.3386±0.0054	0.3198±0.0039
XGB	0.9101±0.0005	0.8218±0.0005	0.6904±0.0170	0.5953±0.0028	0.6513±0.00226
NB	0.2773±0.1207	0.1905±0.0471	0.3138±0.0012	0.8906±0.2187	0.0380± 0.0760
RF	0.8996±0.0008	0.9266±0.0030	0.5901±0.0021	0.4329±0.0053	0.5925±0.0040
SSC	0.8894±0.0013	0.6224±0.0231	0.7128±0.0211	0.8571±0.0001	0.6647± 0.0005

**Table 5:** The performance on the 1:10 imbalanced dataset

	ACC	Pre	$F_{1}$	Recall	MCC
DCSE	**0.9822±0.0017**	**0.8952±0.0012**	**0.9033±0.0002**	**0.9115±0.0102**	**0.8935±0.0080**
DeepPPI	0.9165±0.0093	0.2526±0.3094	0.2238±0.0741	0.20093±0.0461	0.2099±0.2572
DNN-XGB	0.9668±0.0004	0.8903±0.0022	0.7994±0.0123	0.7255±0.0038	0.7865±0.0031
KNN	0.8995±0.0007	0.3864±0.0073	0.2404±0.0013	0.1749±0.0129	0.2127±0.0101
XGB	0.9444±0.0003	0.8173±0.0013	0.6225±0.0201	0.5028±0.0045	0.6149±0.0030
NB	0.2933±0.2207	0.2105±0.0471	0.3037±0.1221	0.8932±0.1186	0.0300±0.0600
RF	0.9375±0.0002	0.9355±0.0027	0.4962±0.0013	0.3377±0.0036	0.5411±0.00231
SSC	0.8883±0.0212	0.4422±0.0013	0.5702±0.1072	0.8616±0.1009	0.5601±0.0312

### Performance on another species

To further verify the generalization of the model, we utilize S. cerevisiae-benchmark dataset as an independent test dataset, which comes from previous work [28] and contains 11,188 protein pairs including 5594 positive, 5594 negative pairs. The protein pairs with sequence identities of 40% or higher were removed. To select the negative there are two principles: (1) the number of negative pairs is equal to the number of positive pairs; and (2) the two proteins in a negative pair do not share subcellular localization. We follow the 8:2 division ratio to divide it into a training set and a test set. Note that no hyperparameter needs to be adjusted so the validation set is needless. Because of the natural differences between human proteins and cerevisiae proteins, only training on human proteins and then testing on cerevisiae cannot fully reflect the performance of the model. In order to better prove the generalization of the model, we freeze all the parameters except for the prediction layer and just train the prediction layer in the training set of cerevisiae. The result is shown in Table 6. Our model also obtains excellent performance on the cerevisiae species by training the feature extraction layer in the homosapiens dataset only, which reflects our model has good generalization.

**Table 6:** The performance on the S.cerevisiae-benchmark

	ACC	Pre	$F_{1}$	Recall	MCC
	0.9602±0.0001	0.9856±0.0002	0.9591±0.0013	0.9340±0.0100	0.9217±0.0016

## Conclusion

In this paper, we propose the DCSE model to predict PPI based on protein sequence. A comparison with seven other models is made and the result shows that our model achieves the best results on the same dataset. Through our experiments, we found that NLP-based encoding methods can achieve good results on PPI tasks. MCN and MBC extract protein sequence features from local and global perspectives and these two feature extraction layers are based on siamese and ensemble network structure. Siamese-based network structure can keep the features consistent and ensemble-based network structure can effectively improve the accuracy of the model.

Even though our model achieves good results, there are still some shortcomings can be improved. Our model only uses sequence features instead of structural features, and the structure features usually contains more information. We look forward to future work to extract protein features from the perspective of protein structure.

## Methods

**Fig. 7:**
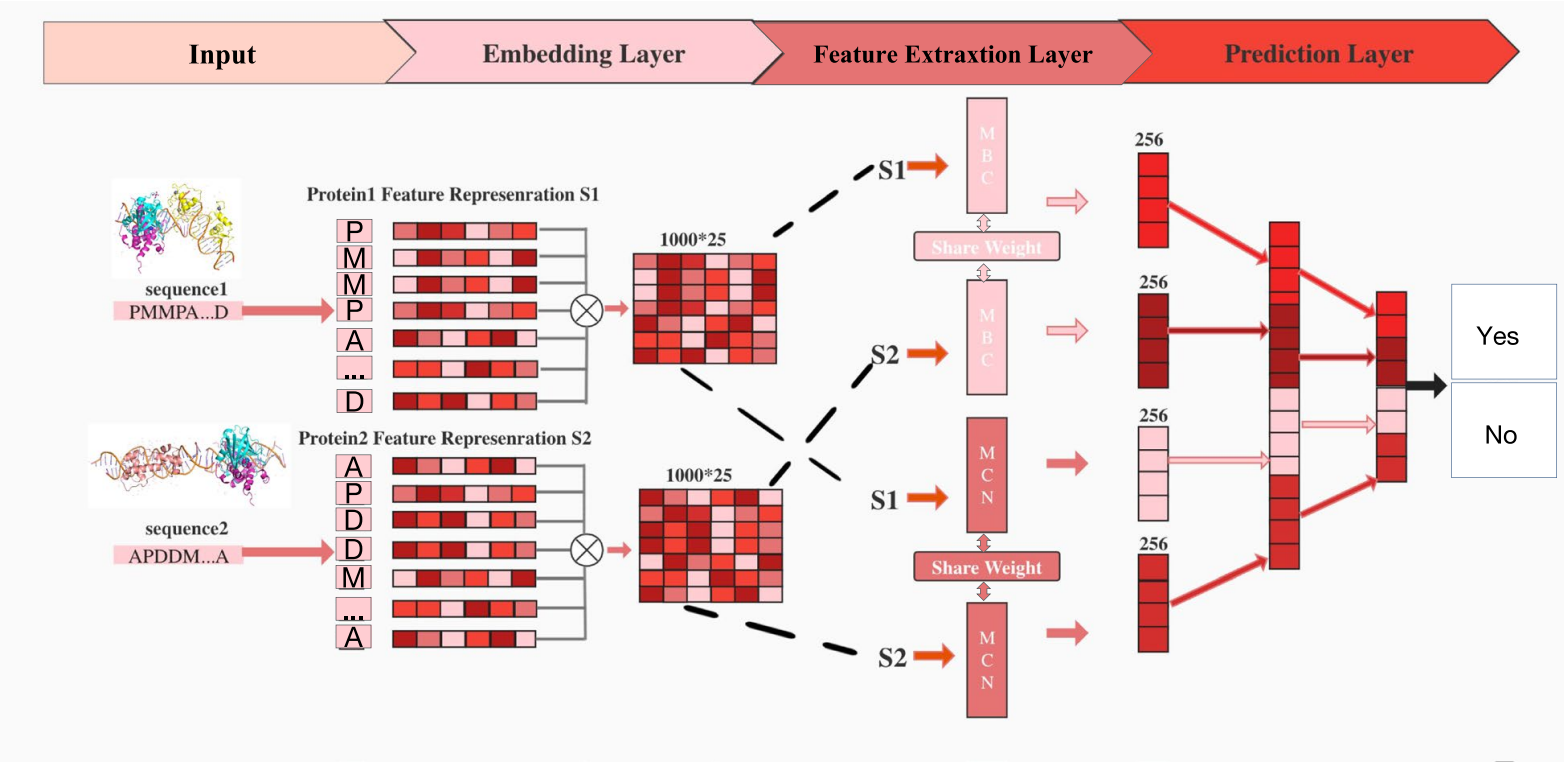
DCSE consists of three main parts, which are the embedding layer, the feature extraction layer, the prediction layer. The input of our model is protein sequence1 and protein sequence2. Both the sequence is truncated the first 1000 amino acids if the sequence is more than 1000 or padded to 1000 if the sequence is less than 1000. In the embedding layer, each amino acid is mapped into a 25-dimensional vector so each protein can be represented by a 1000*25 feature matrix(S1, S2). Both the S1 and S2 are then fed into the siamese-based feature extraction layer. Then the output of the feature extraction layer is concatenated together and put into the prediction layer which finally gives the predictive result of our model

The proposed architecture of DCSE is shown in Fig. 7, which consists of an embedding layer, two feature extraction layers (MCN, MBC), and a prediction layer. Specifically, the embedding layer aims to turn amino acid sequences with different lengths into the same dimension feature matrix. The feature extraction layer is introduced to further extract useful local features and global features of protein sequence. Finally, the outputs of the extraction layer are combined and put into the prediction layer to predict whether these two proteins would interact with each other. A detailed description of each part of DCSE is made in the following section.

### Embedding layer

**Fig. 8:**
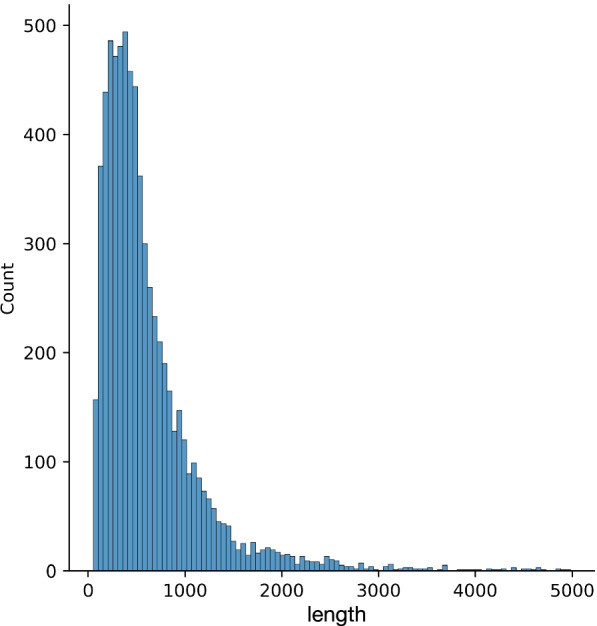
The length distribution of our dataset

Regarding each amino acid as a word, we can map each amino acid into the N-dimensional space to encode the protein sequence. Specifically, before inputting the sequence into our model, our preprocessing is to convert each amino acid into a token, and each token is mapped to the N-dimension initialized with learnable parameters from a normal distribution. We fix the length of protein sequence with 1000. We analyze the length distribution which is shown in Fig. 8, and about 86% of the protein sequence length is less than 1000, so we truncate the first 1000 amino acids to reserve the majority of the sequence information. If the length is greater than 1000, we truncate the first 1000 amino acids, and if the length is less than 1000, we perform the padding operation. Assuming a protein sequence with 950 amino acids, when the raw sequence is input into the embedding layer, it is first transformed to a feature matrix with a size of 950*N. Then we use a 50*N matrix with all the values being set to 0, which is concatenated with the 950*N matrix to compose a 1000*N matrix. By the above encoding process, a protein feature matrix with a fixed dimension of 1000*N is obtained. We test N to be 20,25,30 and find that when N is set to 25, our model gets the best performance.

It is worth mentioning that in Nikhil et al’s work [12], they treat K consecutive amino acids as the smallest unit (K-mer) and map the K-mers together to an N-dimensional space. Specifically, in the MVMRQAGP protein sequence, if K is 2, then MV, VM, MR, RQ, QA, AG, GP are the smallest units, and these smallest units which constitute the representation of protein sequence will be mapped to the N-dimensional space. We also test the different values of K in [1,2,3,4] and find that when K is set to 1 our model obtains the best performance.

### Feature Extraction Layer

The sequence of amino acid residues is determined by the sequence of the genetic code on the gene, and adjacent amino acids are linked by peptide bonds, so the local structure of protein sequence is important. Besides, PPI occurs in 3D space and the 3D structure of protein is completed through the folding of amino acid chains. Non-adjacent amino acids in the amino acid sequence may also be adjacent in the 3D space through folding, resulting in mutual influence. Therefore, the features of non-adjacent amino acids are also important. In this work, we design two channels to extract the local and global features of protein sequence in parallel. One channel for local feature extraction is Multilayer Convolutional Neural Network (MCN). The other one for global feature extraction is Multilayer Bidirectional Gated Recurrent Unit with Convolutional Neural Networks (MBC). Convolutional Neural Networks (CNN) and Gate Recurrent Unit (GRU) all have shown good performance for extracting protein features in recent studies [29, 30].

We apply CNN to learn the features of adjacent amino acids and use GRU to learn the global sequence features. The major component of MBC is GRU, whose special advantage is to capture the long-distance dependence of non-adjacent amino acids. GRU integrates all the features of each amino into the output matrix after some processes, so the output of GRU contains the entire sequence information of the given protein. The main part of MCN is 1D CNN, which can learn from the raw sequence data directly and map amino acid features to a higher-dimensional space so that we can obtain more complicated biological information. In addition, by adjusting the parameter kernel and stride [31], 1D CNN can put more attention to the local feature so we can obtain more local protein features. After getting the local feature and global feature from two channels respectively, we concatenate the output of MCN and MBC together to obtain the final prediction through prediction layer.

#### MCN Layer

MCN includes a four-layer CNN, and each layer includes a 1D CNN, a BatchNorm layer, a Max-pooling layer. The input of MCN is the protein feature matrix $S \in R^{1000 * N}$ . Finally, the output of the four-layer CNN is input to the flatten layer and the liner tranformation layer, and the final output is a 256-dimensional vector.

CNN has advantages in local perception, which can ensure that the convolution kernel can fully consider local features [32]. 1D convolution is majorly used where the input is sequential such as text or audio [33], which is very suitable for protein sequences. The formula for the operation of the 1D convolution is defined as follows:

6 $$\begin{aligned} O(i,\ j)=f\left( \sum\limits _{m=0}^{L r-1} \sum\limits_{n=0}^{L c-1} x(i+m,\ j+n) * w(m,\ n)+b\right) \end{aligned}$$

7 $$\begin{aligned} f(x)=\left\{ \begin{array}{lr} x &{} x>0 \\ 0.3 * x &{}x \le 0 \end{array}\right. \end{aligned}$$

Where $\mathrm {O}(\mathrm {i},\ \mathrm {j})$ represents the convolution operation result of the i-th row and j-th colomn located in the S. *f(x)* is the LeakyRelu activation function with setting negative slope=0.3. *x* is the input of 1D CNN, and *w(m, n)* is the value of the m-th row and the n-th column in the convolutional kernel matrix. Lr and Lc represent the row height and column width of the convolution kernel matrix (w) respectively.

**Fig. 9:**
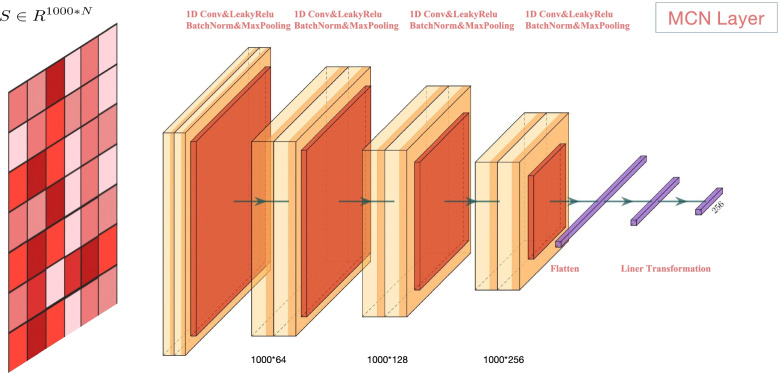
MCN includes 4 layers Conv1D, BatchNorm layer, Max-pooling layer. The output of MCN is a 256-dimensional vector

The BatchNorm layer and Max-pooling layer are added after the 1D CNN is to standardize the output of the 1D CNN and avoid overfitting of the model. The MCN layer is shown in Fig. 9.

#### MBC Layer

MBC Layer is composed of a 1D CNN, and a bidirectional two-layer GRU. The input of MBC is the protein feature matrix $S \in R^{1000 * N}$. 1D CNN aims to map the dimension of the feature to a higher dimension in the MBC layer. The output of bidirectional two-layer GRU is finally input into the flatten layer and liner transofrmation layer. Finally, the output of MBC is a 256-dimensional vector.

Both LSTM and RNN are classic models in the NLP field. RNN has gradient explosion and gradient disappearance phenomena [34], which makes the algorithms unsuitable to handle long sequences. The model structure of LSTM is complicated [35], which includes an input gate, forget gate, and output gate, so the training time is too long. Therefore, we use the GRU-based model. GRU is a simplified version of LSTM and a special form of RNN. Unlike the complex network structure of LSTM, GRU combines the input gate and forgetting gate into an update gate, so the model parameters are reduced. One of the cores of GRU is gate mechanisms, which can effectively alleviate the time-consuming problem of LSTM. GRU has two inputs, one is the current amino acid feature, and the other is the hidden feature of previous amino acid. The processing flow of the two inputs is shown below.

8 $$\begin{aligned} r_{t}=\sigma \left( W_{i r} S_{t}+b_{i r}+W_{h r} h_{(t-1)}+b_{h r}\right) \end{aligned}$$

9 $$\begin{aligned} z_{t}=\sigma \left( W_{i z} S_{t}+b_{i z}+W_{h z} h_{(t-1)}+b_{h z}\right) \end{aligned}$$

10 $$\begin{aligned} n_{t}=\tanh \left( W_{i n} S_{t}+b_{i n}+r_{t} *\left( W_{h n} h_{(t-1)}+b_{h n}\right) \right) \end{aligned}$$

11 $$\begin{aligned} h_{t}=\left( 1-z_{t}\right) * n_{t}+z_{t} * h_{(t-1)} \end{aligned}$$

Where $h_{t}$ is the hidden feature of t-th amino acid. $x_{t}$ is the feature of t-th amino acid. $h_{t-1}$ is the hidden feature of the (t-1)-th amino acid in GRU or the initial hidden state at time 0. $r_{t}$ ,$z_{t}$ ,$n_{t}$ are the reset, update, and new gate, respectively. $\sigma$ is the sigmoid function, * is the hadamard product, and $b_{ir}$ ,$b_{hr}$ ,$b_{iz}$ ,$b_{hz}$ ,$b_{in}$ ,$b_{hn}$ are the bias.

**Fig. 10:**
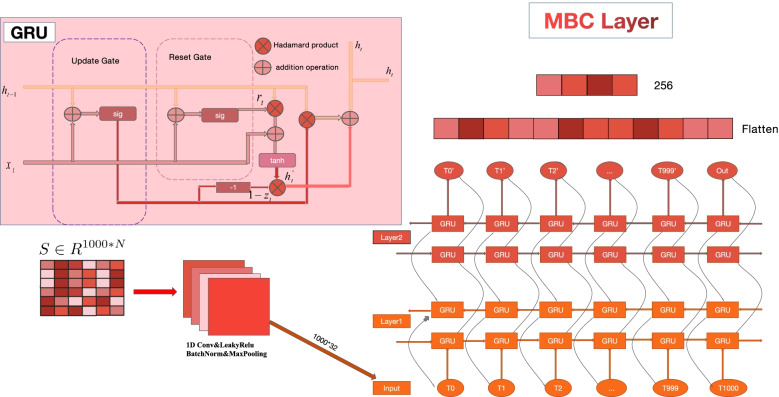
The inner structure of GRU and our MBC layer. The output of MBC is a 256-dimensional vector

Although GRU can obtain good results on PPI tasks, the drawback is that it extracts sequence feature from one side to another side. When the sequence is too long, the phenomenon of Long-Term Dependencies [36] will appear and affect the predictive accuracy of the model. Bidirectional GRU extracts feature from the left and the right of the sequence at the same time, which can solve the problem of Long-Term Dependencies. The processing flow of Bidirectional Gated Recurrent Unit (BIGRU) and standard GRU is almost the same, but the difference is that $h_{t}$ has two parts from left to right and from right to left. The basic GRU and BIGRU are shown in Fig. 10 and the calculation method of BIGRU can be expressed as:

12 $$\begin{aligned} \overrightarrow{H_{t}}=G R U\left( {S}_{t},\ \overrightarrow{H}_{t-1}\right) \end{aligned}$$

13 $$\begin{aligned} \overleftarrow{H_{t}}=G R U\left( {S}_{t},\ \overleftarrow{H}_{t-1}\right) \end{aligned}$$

14 $$\begin{aligned} \mathrm {H}_{\mathrm {t}}=\mathrm {p}_{\mathrm {t}} \overrightarrow{\mathrm {H}}_{\mathrm {t}}+\mathrm {q}_{\mathrm {t}} \overleftarrow{H}_{\mathrm {t}}+\mathrm {b}_{\mathrm {t}} \end{aligned}$$

Where GRU(S, H) indicates the mentioned GRU network to process the sequence from one side to another side, $S_{t}$ indicates the t-th amino acid feature, $H_{t-1}$ is the output feature of the (t-1)-th amino acid, and $b_{t}$ is the bias of the state of the hidden layer.

#### Siamese network

Siamese network was first proposed for signature and image matching [32], in which different inputs are processed by the same network. Unlike other structures, the weights between the same networks are shared, and the parameters of the two networks are updated simultaneously. In the field of face recognition [37, 38], the input is the face picture information of two people, and the two networks respectively extract different parts of the two face pictures. Similar ideas can also be used in PPI work. Previous work [5] put two protein sequence features into two same models which share weights. Specifically, to capture complex relationship between two proteins they [5] employ a siamese CNN architecture with two identical CNN sub-networks that share the same parameters for a given pair of protein profiles. The reason for using such a structure in PPI is because extracting a pair of protein features in a model at the same time instead of putting a pair of sequences into two same models with two sets of parameters can keep features consistent.

**Fig. 11:**
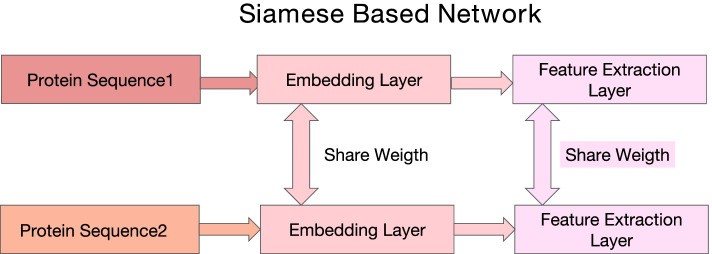
The simple example of siamese-based network

Inspired by previous work [5], our Embedding Layer, MCN-Layer, and MBC-Layer all use the structure of the siamese network. They share weights and have the same network structure. The embedding layer is designed as the siamese network because it can ensure the protein feature follow the same distribution, which can help the model easily extract more deeper information. The reason why the MCN-Layer and MBC-Layer are designed as the siamese netowrk are same, which is to guarantee the feature keeping consistent so the model can easily extract the effective and useful feature. The simple network is shown in Fig. 11.

#### Ensemble network

Ensemble network, in simple terms, is to combine multiple models together. This method often achieves better performance than any single component learning algorithm in the predictive task [22, 23]. In our method, MCN and MBC can be regarded as two models respectively. Through our test, combining the two models together is better than using one of them alone. Different channels aim to extract different features of protein sequence, which involves local feature and global feature. In order to balance the contribution of two channels, we introduce a hyperparameter $\beta$ to give weight to the outputs of the two channels. Specifically, assuming that the weight of MBC is $\beta$ , so the weight of MCN is 1-$\beta$ . The feature matrix of protein1 is S1, and the feature matrix of protein2 is S2. After the processing of MBC and MCN, each output of different channels is multiplied with the corresponding weight. The calculation of the protein pair features is as follows:

15 $$\begin{aligned} X=[(1-\beta ) *[M C N(S 1),\ M C N(S 2)],\ \beta *[M B C(S 1),\ M B C(S 2)]] \end{aligned}$$

Where X indicates the final features of the protein pair. [,] means the concatenation operation.

### Prediction layer and loss function

After the feature extraction of the protein pairs, we get a 1024-dimensional vector. The feature vector contains four parts, which are the features of protein sequence S1 extracted by MBC and MCN separately, and the features of protein sequence S2 extracted by MBC and MCN separately. The features of each part is 256-dimensional vector. In the prediction layer, multi-layer perceptron (MLP) is used to decide whether these two proteins will interact.

16 $$\begin{aligned} y=\mathbf {W}_{2}\left( \text{ LeakyRelu } \left( \mathbf {W}_{1} X+b\right) \right) \end{aligned}$$

Where $W_{1}\in R^{512 * 1024}$ and $W_{2}\in R^{ 2*512 }$, $X \in R^{1024*1}$, $y \in R^{2*1}$ , *b* is the bias. The out put of prediction layer is 2-dimensional vector, indicating the probability of non-interaction and interaction separately.

We utilize cross entropy loss as our loss function, and it is calculated as follows:

17 $$\begin{aligned} Loss =-\frac{1}{N} \sum\limits_{i=1}^{N} y_{i} \cdot \log \left( p\left( y_{i}\right) \right) +\left( 1-y_{i}\right) \cdot \log \left( 1-p\left( y_{i}\right) \right) \end{aligned}$$

Where N means the total samples, $y_{i}$ means the probability that the i-th protein pair interacts each other.

## Supplementary Information

**Additional file 1.**

## Data Availability

The model implemented by Pytorch can freely visit in source code, and the dataset can be freely found in StringV11.

## Abbreviations

PPI: Protein-protein interaction
DCSE: Double-Channel-Siamese-Ensemble
MBC: Multilayer Bidirectional Gated Recurrent Unit with Convolutional Neural Networks
MCN: Multilayer Convolutional Neural Network
PSSM: position-specific scoring matrix
CT: Conjoint Triads
AC: Auto-Covariance
LD: Local Descriptor
NLP: Natural language processing technology
K-mer: K consecutive amino acids
CNN: Convolutional Neural Networks
GRU: Gate Recurrent Unit
TP: True positives
TN: True negatives
FP: False positives
FN: False negatives
NB: Naive Bayes
KNN: K-nearest neighbors
RF: Random Forest
XGB: XGBoost
DNN: Deep-neural-network
AAC: Amino Acid Composition
DN: DipeptideComposition
QSOD: Quasi-sequence-order
CT-AC-LD: Conjoint Triads+Auto-Covariance+Local Descriptor
