# Supplementary material for: DCSE:Double-Channel-Siamese-Ensemble model for protein protein interaction prediction
Source: BMC Genomics. 2022 Aug 4;23:555. doi: 10.1186/s12864-022-08772-6 (PMC9351149; doi:10.1186/s12864-022-08772-6)
Supplement: Supplementary file 1 — Additional file 1. [file 12864_2022_8772_MOESM1_ESM.pdf]

Supplementary Table1:The detailed information on DCSE

	Accuracy	Precision	Recall
DCSE	0.9312	0.9092	0.9488
	0.9300	0.9094	0.9442
	0.9309	0.9092	0.9442
	0.9292	0.9088	0.9461
	0.9303	0.9089	0.9427
Average	0.9303	0.9091	0.9452
standard deviation	0.0007	0.0002	0.0021
confidence	0.0006	0.0002	0.0018
upper bound(average+confidence)	0.9309	0.9093	0.9470
lower bound(average-confidence)	0.9297	0.9089	0.9434

Supplementary Table2:The detailed information on DNN-XGB

	Accuracy	Precision	Recall
DNN-XGB	0.9281	0.9240	0.9213
	0.9287	0.9257	0.9297
	0.9300	0.9261	0.9252
	0.9296	0.9216	0.9247
	0.9276	0.9241	0.9240
Average	0.9288	0.9243	0.9250
standard deviation	0.0009	0.0016	0.0027
confidence	0.0008	0.0014	0.0024
upper bound(average+confidence)	0.9296	0.9257	0.9274
lower bound(average-confidence)	0.9280	0.9229	0.9226

Supplementary Table2:T-test between DCSE and DNN-XGB

Accuracy	0.0301
Precision	0.0000
Recall	0.0000
F1	0.0377
MCC	0.3127

Supplementary Table4:The detailed information on DeepPPI

DeepPPI	Accuracy	Precision	F1
Average	0.8147	0.7815	0.8118
standard deviation	0.0011	0.0086	0.0189
confidence	0.0010	0.0075	0.0166
upper bound(average+confidence)	0.8157	0.7890	0.8284
lower bound(average-confidence)	0.8137	0.7740	0.7952

Supplementary Table5:The detailed information on KNN

KNN	Accuracy	Precision	F1
Average	0.7300	0.7157	0.7131
standard deviation	0.0039	0.0039	0.0009

confidence	0.0034	0.0034	0.0008
upper bound(average+confidence)	0.7334	0.7191	0.7139
lower bound(average-confidence)	0.7266	0.7123	0.7123

Supplementary Table6:The detailed information on XGB

XGB	Accuracy	Precision	F1
Average	0.8315	0.8345	0.8180
standard deviation	0.0026	0.0031	0.0003
confidence	0.0023	0.0027	0.0003
upper bound(average+confidence)	0.8338	0.8372	0.8183
lower bound(average-confidence)	0.8292	0.8318	0.8177

Supplementary Table7:The detailed information on NB

NB	Accuracy	Precision	F1
Average	0.5016	0.5044	0.6443
standard deviation	0.0586	0.0642	0.0010
confidence	0.0514	0.0563	0.0009
upper bound(average+confidence)	0.5530	0.5607	0.6452
lower bound(average-confidence)	0.4502	0.4481	0.6434

Supplementary Table8:The detailed information on RF

RF	Accuracy	Precision	F1
Average	0.8263	0.8720	0.8011
standard deviation	0.0026	0.0035	0.0013
confidence	0.0023	0.0031	0.0011
upper bound(average+confidence)	0.8286	0.8751	0.8022
lower bound(average-confidence)	0.8240	0.8689	0.8000

Supplementary Table9:The detailed information on SSC

SSC	Accuracy	Precision	F1
Average	0.7722	0.7899	0.7383
standard deviation	0.0013	0.0061	0.0002
confidence	0.0011	0.0053	0.0002
upper bound(average+confidence)	0.7733	0.7952	0.7385
lower bound(average-confidence)	0.7711	0.7846	0.7381
lower bound(average-confidence)	0.7709	0.7838	0.7381
